# Supplementary material for: Effects of Different Dietary Carbohydrate Sources on the Meat Quality and Flavor Substances of Xiangxi Yellow Cattle
Source: Animals (Basel). 2022 Apr 28;12(9):1136. doi: 10.3390/ani12091136 (PMC9105694; doi:10.3390/ani12091136)
Supplement: Supplementary file 1 [file animals-12-01136-s001.zip › animals-1668184-supplementary.pdf]

Supplementary Figure

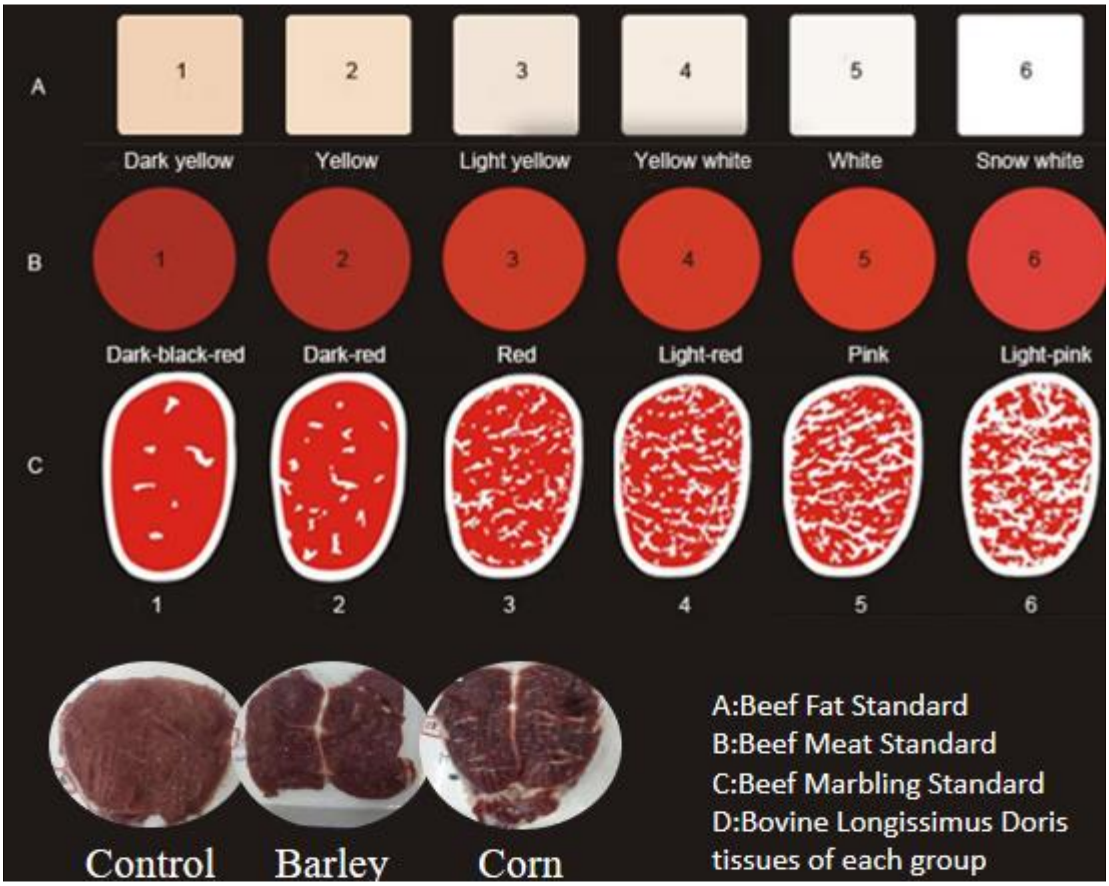

Figure S1: SEUROP classification according to the official Japanese Meat Grading Standards which have been adopted by the Chinese beef cattle industry along with other meat grading standards.
